# Beyond the Skin: Granulomatous Uveitis Linked to Active Tattoo Inflammation

**DOI:** 10.7759/cureus.102255

**Published:** 2026-01-25

**Authors:** Poliana de Jesus Araujo, Marlos Henrique Sousa de Oliveira Junior, Rodrigo A Torres, Ricardo Danilo C Oliveira, Paulo C Fontana

**Affiliations:** 1 Ophthalmology, Universidade Federal da Bahia, Salvador, BRA

**Keywords:** ophtalmology, sarcoidosis, tattoo., tattoo granulomas with uveitis (tagu), uveitis

## Abstract

Tattoo-associated granulomatous uveitis is an uncommon inflammatory reaction in which ocular findings accompany immune responses at tattoo sites. We report a young woman who presented with bilateral visual symptoms along with redness and scaling over longstanding black-ink tattoos. Infectious and systemic causes were excluded through comprehensive evaluation, and skin biopsy confirmed granulomatous inflammation with exogenous pigment. The patient improved with systemic and topical corticosteroids. This case highlights the importance of recognizing tattoo-related inflammatory responses as a potential cause of uveitis when no clear etiology is identified.

## Introduction

Tattoo-associated granulomatous uveitis (TAGU) is an uncommon inflammatory condition in which intraocular inflammation occurs alongside granulomatous reactions in tattooed skin. This entity is most commonly referred to as TAGU and represents a rare immune-mediated reaction triggered by tattoo pigments. Although cutaneous complications related to tattoos-such as infiltration, nodules, and scaling-are relatively well described, their association with uveitis remains infrequent and often underrecognized.

The first description of this association dates back to 1952, when Lubeck and Epstein reported patients who developed ocular inflammation concurrently with granulomatous changes within tattoos [1]. Since then, TAGU has been increasingly reported, though its exact pathophysiology remains incompletely understood. Two main hypotheses have been proposed. The first suggests a delayed hypersensitivity reaction to tattoo pigments, which may contain heavy metals and organic compounds with antigenic potential, leading to a cell-mediated immune response affecting both the skin and the eye. The second hypothesis proposes that chronic exposure to pigment antigens may induce a systemic granulomatous reaction resembling a limited form of sarcoidosis [2].

Clinically, distinguishing TAGU from sarcoidosis can be challenging, as both conditions may present with non-caseating granulomas on histopathology. However, TAGU typically lacks systemic involvement at presentation, and the identification of exogenous pigment within granulomas, combined with the absence of pulmonary or lymphatic disease, favors a tattoo-related etiology. Despite the increasing prevalence of tattoos worldwide, affecting approximately one-quarter of adults in the United States, immune-mediated reactions such as TAGU remain rare and are likely underdiagnosed [3]. A comprehensive literature review spanning more than six decades identified fewer than 40 reported cases, most commonly associated with black-ink tattoos and younger patients [4]. 

TAGU may present as anterior uveitis, intermediate uveitis, or panuveitis, frequently with bilateral involvement and concurrent inflammatory changes in tattooed skin, such as erythema, desquamation, or swelling. Corticosteroid therapy remains the mainstay of treatment, though some patients require prolonged immunosuppression or tattoo removal in recurrent cases [4-5].

This case highlights the importance of considering tattoo-related granulomatous inflammation in the differential diagnosis of uveitis in young patients with no identifiable systemic disease, particularly when ocular symptoms coincide with inflammatory changes in longstanding tattoos. Early recognition of this association may prevent diagnostic delays and unnecessary investigations while guiding appropriate management.

## Case presentation

**Figure 1:**
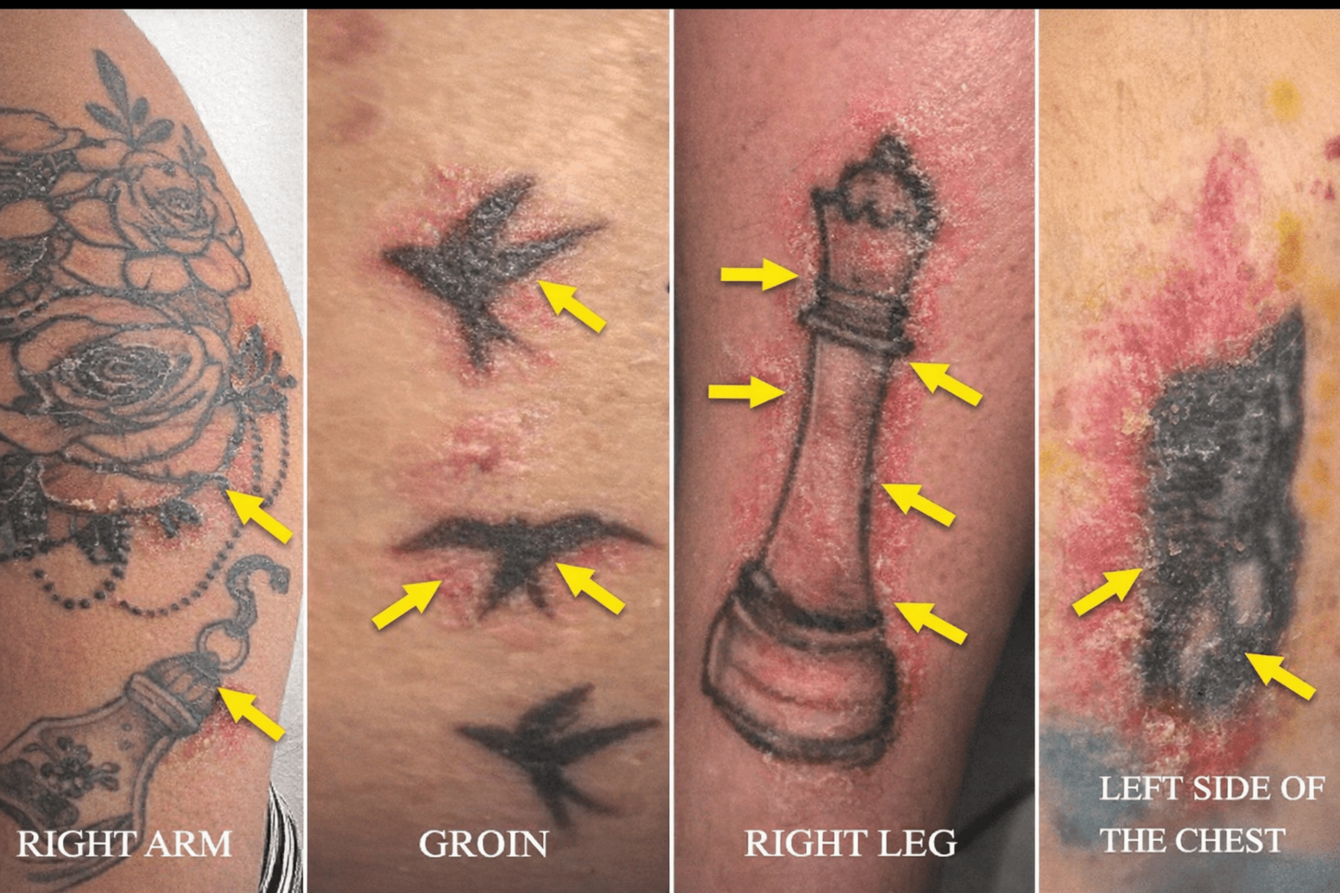
Clinical photograph showing inflammatory changes in a large black-ink tattoo. Arrows highlight areas of erythema, skin thickening, and scaling at the margins of the tattoo, consistent with active granulomatous inflammation.

A 21-year-old Brazilian female presented with bilateral hyperemia and blurred vision, more intense in the right eye, with symptom onset approximately one month before presentation. Concomitant with the ocular symptoms, she developed cutaneous manifestations characterized by intense redness and scaling at the margins of multiple tattoos on her upper limbs (Figure 1). The patient reported having a total of 10 tattoos, all performed between 2019 and 2022, and noted that the interval between her most recent tattoo and the day of clinical evaluation was approximately two years, with no prior local or systemic inflammatory reactions before the onset of symptoms.

**Figure 2:**
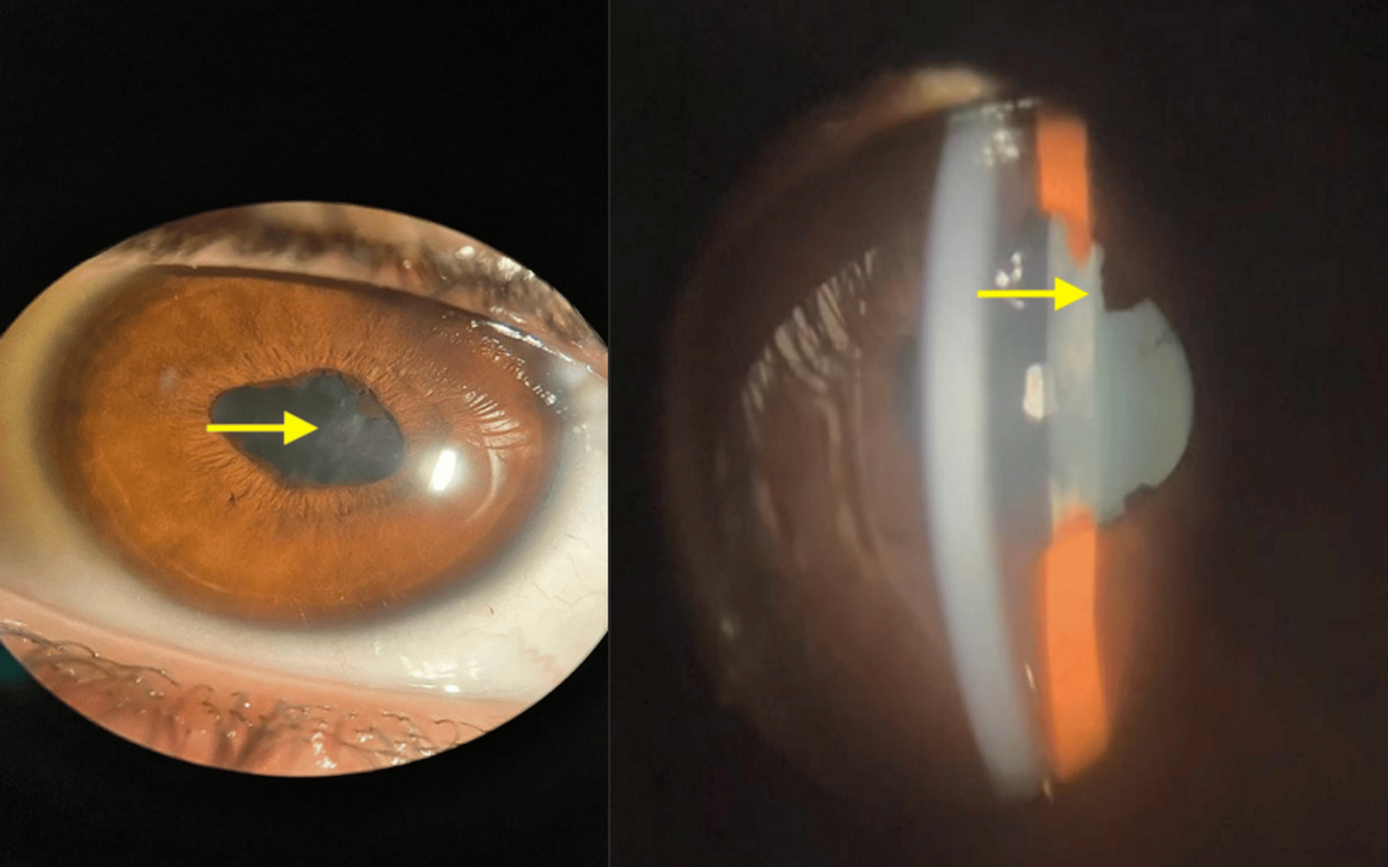
Slit-lamp photograph of the right eye demonstrating posterior synechiae and anterior lens opacity (arrows), consistent with granulomatous anterior uveitis.

**Figure 3:**
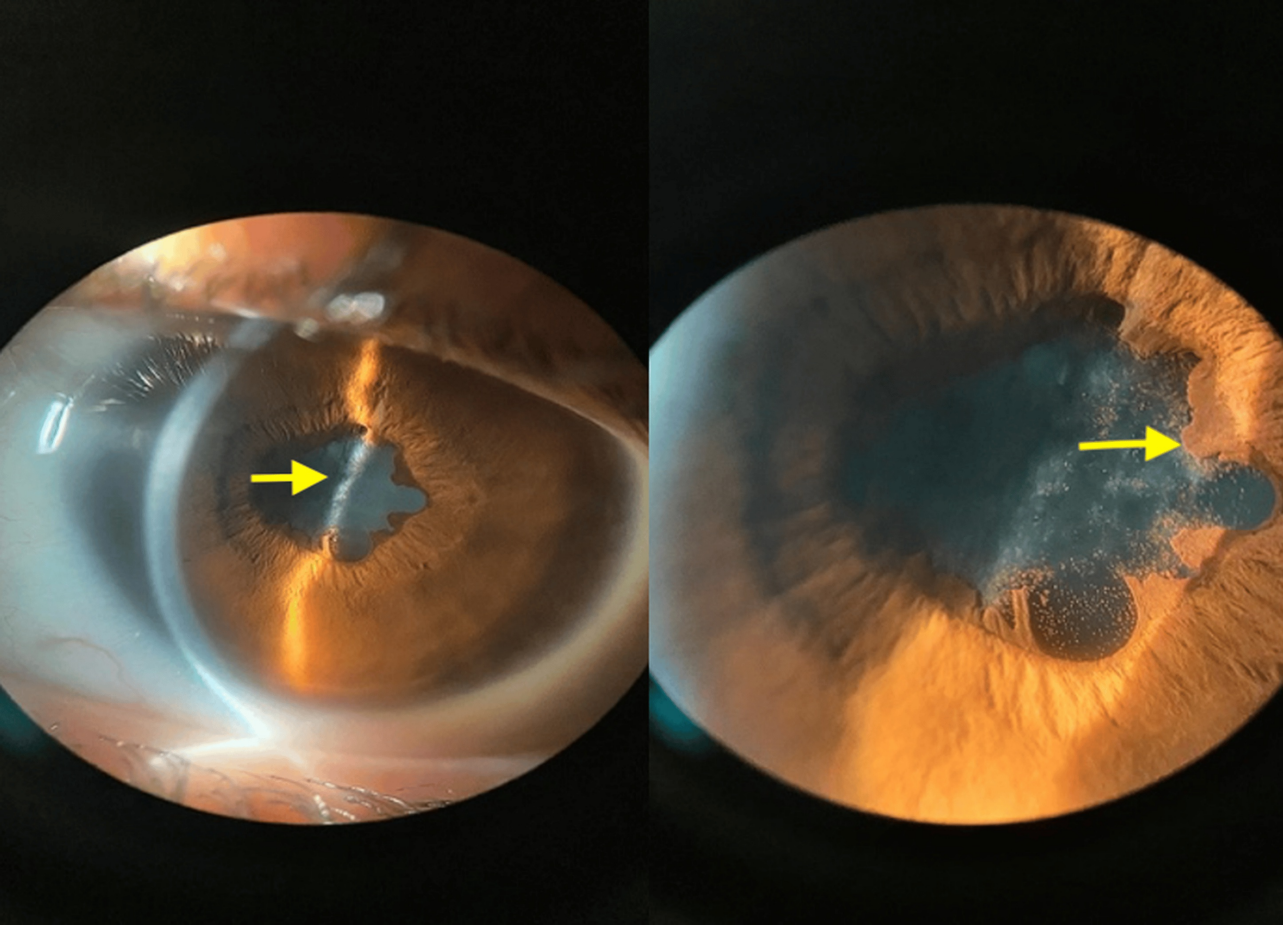
Slit-lamp photograph of the left eye showing posterior synechiae and lens opacity (arrows), supporting bilateral anterior segment involvement.

**Figure 4:**
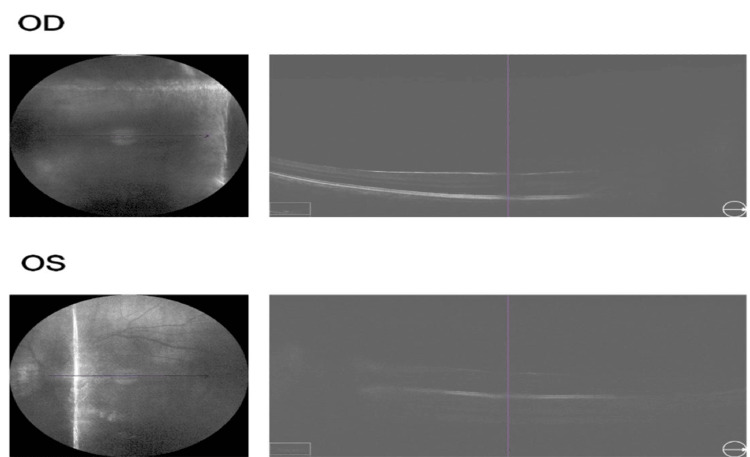
Optical coherence tomography of the macula showing media opacity and retinal thickening in the macular region.

On ophthalmological examination, the patient presented visual acuity of 20/200 in the right eye and 20/80 in the left eye, according to the Snellen Chart, and intraocular pressure of 16 mm/Hg in both eyes. Biomicroscopy showed ocular hyperemia, vascular congestion, posterior synechiae, corectopia, anterior chamber reaction +2, and anterior lens opacity in both eyes (Figures 2-3). Fundoscopy using retinal mapping showed vitreous opacity, papilledema, and macula with decreased macular brightness. Optical coherence tomography of the macula was performed, with evidence of media opacity and retinal thickening in the macular region (Figure 4).

**Figure 5:**
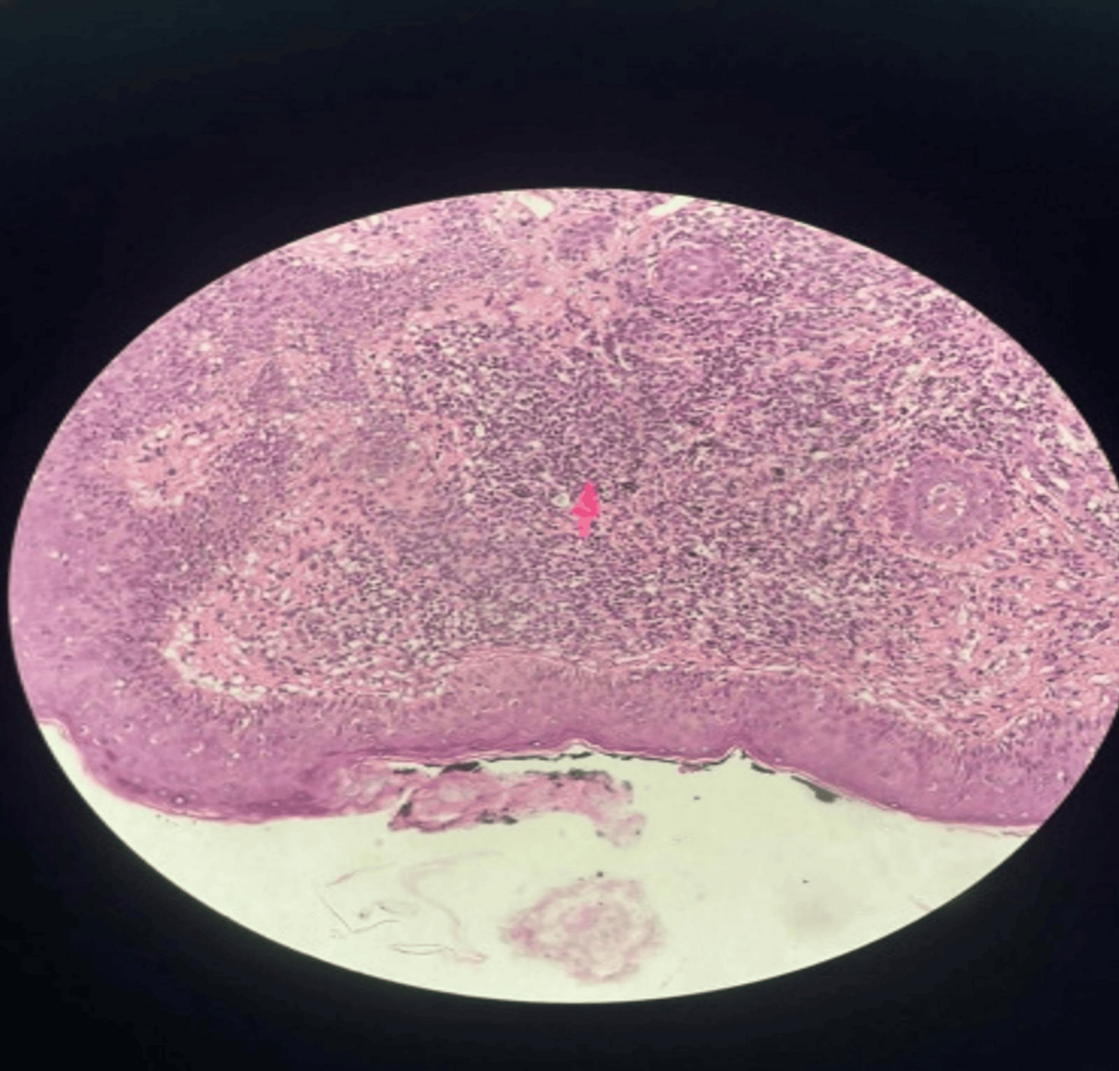
Histopathological examination of the tattooed skin showing non-caseating granulomatous inflammation with exogenous black pigment deposition (arrow) (hematoxylin and eosin stain, original magnification ×40).

Erythrosedimentation rate and C-reactive protein were within the normal range. Infectious causes such as herpes, toxoplasmosis, bartonellosis, toxocariasis, rubella, syphilis, HIV, and cytomegalovirus were ruled out. Autoantibodies such as antinuclear factor and anti-dsDNA antibodies were negative. Serum angiotensin-converting enzyme and lysozyme levels were normal. Her chest tomography showed no abnormalities. The histopathological study of the skin demonstrated a granulomatous process associated with exogenous black pigment (Figure 5). 

Systemic treatment with oral prednisone at an initial dose of 60 mg daily, combined with topical prednisolone acetate 1% eye drops four times daily, was initiated. Progressive improvement of both ocular inflammation and cutaneous lesions was observed over six months of follow-up. At the most recent evaluation, the patient remains asymptomatic while receiving low-dose prednisone (5 mg daily), with no evidence of recurrent inflammatory flares to date.

## Discussion

Patients undergoing evaluation for uveitis should be specifically questioned about the presence of tattoos and any prior episodes of inflammation affecting tattooed skin. The clinical manifestations of tattoo-associated uveitis vary depending on the type and severity of intraocular inflammation. Cutaneous findings often include erythema, edema, thickening, warmth, tenderness, or desquamation over the tattooed area, while ocular signs may involve conjunctival or scleral injection, photophobia, anterior chamber cells and flare, granulomatous keratic precipitates, posterior synechiae, intraocular pressure changes, vitreous cells, vasculitis, choroiditis, or other forms of intraocular inflammation [5-7].

TAGU remains a diagnosis of exclusion. Sarcoidosis must always be carefully considered and thoroughly ruled out, as both entities may present with non-caseating granulomas on histopathology. Distinguishing sarcoidosis from a foreign-body granulomatous reaction to tattoo pigment is often challenging, since histological findings alone are insufficient. Most reported TAGU cases undergo extensive systemic evaluation, and although pulmonary involvement may be absent, the lack of systemic disease combined with the identification of exogenous pigment within granulomas favors a tattoo-related etiology [8-10].

An important feature of TAGU is the often prolonged latency between tattoo placement and the onset of clinical symptoms. Many patients experience a period of apparent immunologic tolerance lasting several years before developing ocular and cutaneous inflammation, as observed in the present case. This delayed presentation supports the hypothesis that chronic exposure to tattoo pigments may lead to gradual immune sensitization rather than an immediate hypersensitivity reaction. Persistent antigenic stimulation may eventually exceed an immunologic threshold, resulting in localized or systemic granulomatous inflammation [2-16].

Systemic corticosteroid therapy was initiated in this patient due to the presence of bilateral panuveitis associated with active granulomatous inflammation at multiple tattoo sites, aiming to control both ocular and cutaneous immune-mediated disease simultaneously. The favorable clinical response observed is consistent with previous reports describing good responsiveness of TAGU to immunosuppressive therapy, although optimal treatment duration and tapering strategies remain individualized [8,13,15,16].

This report is limited by its single-patient design, which precludes definitive conclusions regarding causality and limits generalizability. Nevertheless, the detailed chronological documentation, comprehensive exclusion of infectious and systemic granulomatous diseases, and histopathological confirmation of pigment-associated granulomatous inflammation provide clinically relevant insight into a rare and likely underrecognized condition.

## Conclusions

TAGU can present as anterior, intermediate, or panuveitis and may follow a variable clinical course, sometimes assuming a chronic or recurrent pattern. In the present case, bilateral panuveitis, biopsy-proven granulomatous inflammation with exogenous pigment deposition, exclusion of infectious and systemic granulomatous diseases, and a favorable response to corticosteroid therapy supported the diagnosis. Although the patient remains on low-dose systemic corticosteroids and long-term prognosis is uncertain, this report reinforces that TAGU should be included in the differential diagnosis of both acute and chronic uveitis, particularly in younger patients with inflammatory changes in longstanding tattoos. Importantly, this condition should not be considered restricted to panuveitis, as it may present in any anatomical form of uveitis. Careful history-taking and long-term ophthalmologic follow-up are essential for timely diagnosis and optimal management.
